# Analysis of Ionic Domains on a Proton Exchange Membrane Using a Numerical Approximation Model Based on Electrostatic Force Microscopy

**DOI:** 10.3390/polym13081258

**Published:** 2021-04-13

**Authors:** Byungrak Son, JaeHyoung Park, Osung Kwon

**Affiliations:** 1 Division of Energy Technology, DGIST, Daegu 42988, Korea; brson@dgist.ac.kr; 2 Corporate Research Center, HygenPower Co., Ltd., Daegu 42988, Korea; chris@hygenpower.com; 3 Tabula Rasa College, Keimyung University, Daegu 42601, Korea

**Keywords:** electrostatic force microscopy, proton exchange membrane, numerical approximation model, local dielectric constant, ionic domain, surface charge density, PEMFC

## Abstract

Understanding the ionic channel network of proton exchange membranes that dictate fuel cell performance is crucial when developing proton exchange membrane fuel cells. However, it is difficult to characterize this network because of the complicated nanostructure and structure changes that depend on water uptake. Electrostatic force microscopy (EFM) can map surface charge distribution with nano-spatial resolution by measuring the electrostatic force between a vibrating conductive tip and a charged surface under an applied voltage. Herein, the ionic channel network of a proton exchange membrane is analyzed using EFM. A mathematical approximation model of the ionic channel network is derived from the principle of EFM. This model focusses on free charge movement on the membrane based on the force gradient variation between the tip and the membrane surface. To verify the numerical approximation model, the phase lag of dry and wet Nafion is measured with stepwise changes to the bias voltage. Based on the model, the variations in the ionic channel network of Nafion with different amounts of water uptake are analyzed numerically. The mean surface charge density of both membranes, which is related to the ionic channel network, is calculated using the model. The difference between the mean surface charge of the dry and wet membranes is consistent with the variation in their proton conductivity.

## 1. Introduction

Proton exchange membrane fuel cells are a core technology of green energy devices for several reasons. They do not emit carbon dioxide; they can operate continuously under different environmental conditions without change in performance, and they have a relatively high energy conversion efficiency. However, many limitations must be overcome before they can be adopted, such as high cost, low reliability, and a lack of hydrogen gas infrastructure. Solving the low reliability issue is imperative; however, this is difficult because a proton exchange membrane’s reliability is related to its morphological structure.

Proton exchange membranes typically act as proton conductors because of their heterogeneous structures, which is the combination of a hydrophobic backbone with hydrophilic sulfonic acid groups. Sulfonic acid groups create ionic clusters that have an inverted micellar structure and can form a network under hydration. Typically, protons move through the ionic network through vehicle-type and Grotthuss-type mechanisms. In the vehicle-type mechanism, the protons pass into the medium with a solvent. Thus, proton conductivity is related to the solvent diffusion rate. In the Grotthuss-type mechanism, the protons move into the medium by creating and breaking hydrogen bonds without any solvent. In general, these mechanisms are not independent. In the proton exchange membrane, the vehicle-type mechanism is predominant, and the Grotthuss-type mechanism is observed because of the water absorbed into the membrane by hydration [1]. Thus, the structure of the ionic channel network, which exhibits morphological change, is directly related to proton conductivity.

Understanding the morphological structure of Nafion is as important as developing novel membranes. This is because the ability of proton movement to mirror morphological structures such as the ionic channel network is the essential function of proton exchange membranes. Since the 1980s, many research groups have attempted to understand the morphological structure of Nafion [2,3,4]. Gierke et al. introduced a cluster-network model of Nafion based on small-angle X-ray scattering and wide-angle X-ray scattering measurements [5]. According to this model, the ionic channel network is formed by the hydration of ionic clusters, which under dry conditions consist of sulfonic acid groups in a semicrystalline matrix. These ionic clusters are 4 nm diameter spheres in an inverted micellar structure, with a narrow 1 nm channel connecting each cluster. The ionic channel network becomes more widely interconnected as water uptake in the Nafion increases, and the structure becomes more complex as protons move through the network. The most recent of these is Klaus and Chen’s cylindrical water channel model [6], based on simulation studies conducted using existing scattering data. According to Klaus and Chen, cylindrical crystallites of 2–5 nm and cylindrical water channels with a radius of 2–3 nm are formed in the polymer matrix. Each cylindrical water channel increases in size as the volume of water in Nafion increases, and the existence of cylindrical crystallites contributes to the mechanical strength of Nafion. Despite numerous studies on the morphology of Nafion, the structure of the ionic channel network and the proton transport mechanism are still unclear. The morphology of Nafion changes depending on the synthesis process [7]. The morphology varies under hydration and dehydration. In a Nafion-based composite membrane, the morphology is varied with wt% and by using different types of pillar materials [8].

Atomic force microscopy (AFM) can map a specimen’s surface with nanoscale resolution without damage, by using a vibrating tip technique. In addition, it can measure various physical properties such as mechanical, thermal, and electrical properties by using the extended mode [9,10,11]. AFM has been widely used for understanding the morphological characteristics of the proton exchange membrane. Typically, this membrane has a charged/uncharged domain, and its phase separation characteristic is crucial for understanding its characteristics. Thus, conductive AFM techniques, such as electrostatic force microscopy (EFM), current sensing atomic force microscopy, or Kelvin probe microscopy, have attracted attention as efficient means of studying proton exchange membranes. Numerical approaches have been proposed for understanding the morphology of proton exchange membranes; in these, the local charge density and dielectric constant are based on AFM measurements. Thus far, several current-sensing AFM and EFM studies have been conducted [12,13,14,15].

The technique of EFM has great potential for understanding the surface electrical characteristics. It is widely used in studies of the surface charge distribution and dielectric constant of locally charged materials [16,17,18,19]. In EFM, the local charge distribution appears as a phase lag value distribution. For extracting detailed information from the measurements, the decoding process from the recorded phase lag value is required. However, this is difficult because the phase lag occurs owing to the net electrostatic force, which is the summation of all Columbic forces between the tip and the sample surface. Thus, an analytical model is required for EFM measurements, and many models have been suggested. Mélin et al. [16] developed an analytical model for estimating the amount of charge stored on a surface using EFM. They assumed that the tip and sample surface created a parallel-plate capacitor; further, they determined the force gradient of stored charge and dipole–dipole interaction due to the electric field between the tip and the sample surface. By calculating the ratio, the amount of stored charge was derived. Further, they extended this model to consider the tip and sample surface with other capacitor shapes. Han et al. [17] studied the movement and diffusion of natural and injected charges using EFM to understand the interface of a nano-dielectric. They analyzed EFM images using a widely accepted methodical model [18] to explain local charge movement at the SiO_2_/LDPE boundary. In this model, the phase value reflects the net force between the tip and the sample surface and the net force caused by local charge. They used low-density polyethylene (LDPE) as an insulating matrix material to minimize electrical interaction between the tip and the sample surface. Thus, the phase value refers to the amount of local charge, and its movement can be clearly seen. Shen et al. [19] studied the degree reduction of a monolayer graphene oxide (GO) sheet by utilizing electrostatic force spectroscopy; they considered the difference in the dielectric constant of graphene and mica. They assumed that a tip and sample surface can create a parallel capacitor with a dielectric material and derived a capacitive force that includes the dielectric constant between the tip and sample surface.

Previous studies that attempted to understand the local charge distribution and dielectric constant by analyzing EFM signals have obtained remarkable results. EFM signal interpretation is based on characterizing the capacitive force between a tip and sample surface. This capacitive force is due to the electrical interaction between the conductive tip and surface charge of the sample. For calculating the net force, individual electrical interactions that contribute to the net force have to be specified, and this requires a deep understanding of the system. Each suggested analysis based on the capacitive force agreed well with specific systems.

In several studies, EFM signals are used to provide additional morphology information. Thus, the phase value distribution on the surface is used for observing conducting/non-conducting areas of composite membranes [20,21]. A few groups have studied the ionic structure of proton exchange membranes using EFM [22,23]. One such remarkable study is that of Barnes and Buratto [22]. They measured several individual ionic channels of Nafion by using EFM under different bias voltages and analyzed the obtained results using a well-known simple parallel capacitor model. They found particular channel shapes such as connected cylindrical channels, dead-end cylinder channels, and bottleneck channels by characterizing the differences in the EFM signal.

In this study, we derived a numerical approximation model (NAM) for interpreting EFM signals from proton exchange membrane measurements. The subject of our study is similar to the work of Shen et al., whose method focused on understanding locally charged areas, encompassed by non-conducting areas. Further, their approach for analyzing EFM signals was systematic and logical. However, the proton exchange membrane structure is more complicated. The sulfonic acid groups in the membrane, which create ionic clusters, are scattered over the entire surface. Owing to hydration, the ionic clusters are connected with each other, and they create ionic channels. In ionic channels, free charges from ionized sulfonic acid groups or that are externally supplied exist and move. The polarized external electric field caused by applying bias voltage between the tip and surface of the proton exchange membrane causes free charges to coexist near ionic channels. Thus, the capacitive force between the tip and proton exchange membrane simultaneously includes both electrical interactions. To analyze the EFM signal from a proton exchange membrane, the NAM was derived by considering two assumptions. First, the conductive tip and proton exchange membrane surface creates a nanoscopic capacitor, and the geometry of this capacitor can be simplified as a parallel plate. Second, a polarized surface and free charge independently interact with the conductive tip. The electrical interaction of free charge is also considered. NAM considers the sum of two independent electrical interactions: electrostatic force between a conductive tip and polarization surface, and that between a tip and free charges. By considering these two terms, the ionic domain structure can be analyzed. Using this numerical model, we characterize the ionic channel network of proton exchange membranes with different amounts of water uptake. We also extract quantitative information relating the ionic channel network to the proton exchange membrane. Furthermore, we attempt to provide a general model for interpreting changes in the morphology of a proton exchange membrane.

## 2. Experimental Setup and Model Development

Nafion 212 membranes were studied under two different conditions in our experiments. The first membrane, called the dry membrane, was baked in an oven at 80 °C overnight. The second membrane, called the wet membrane, was soaked in water overnight. Before measurement, the dry membrane was exposed to ambient conditions for 2 h, while the wet membrane was soaked in water.

Both membranes were mapped and analyzed systematically in several steps. First, each membrane was scanned at a frequency of 1 Hz as the sample bias voltage was changed from −3 V to 3 V in 1 V intervals by using Park systems XE-150 AFM (Park Systems, Suwon, Korea). Phase images and topography were simultaneously mapped in this step. The mean phase lag value of each image was subsequently calculated and plotted. Finally, these mean phase values were analyzed using an approximation model based on Shen et al.’s study [19].

An electrical interaction occurs when a bias voltage is applied between the tip and the sample surface, as the dielectric sample becomes polarized. The capacitive force that is induced between the tip and the sample surface can be expressed as [24]

(1) $$F=\frac{1}{2}\frac{\partial C}{\partial z}V^{2}$$

where F is the capacitive force, *C* is the capacitance of the space between the tip and the sample, *V* is the applied voltage, and *z* is the distance between the tip and the sample surface. The capacitance of the tip (*C*_tip_), which is modeled as a plate, is [19]

(2) $$C_{\mathrm{Tip}}≅\pi \varepsilon_{0}R_{\mathrm{tip}}^{2}$$

where *R*_tip_ is the radius of the tip and *ε*_0_ is the permittivity of free space. From the capacitance equation, the charge accumulated in the tip is [19]

(3) $$Q_{\mathrm{Tip}}=C_{\mathrm{Tip}}V$$

**Figure 1:**
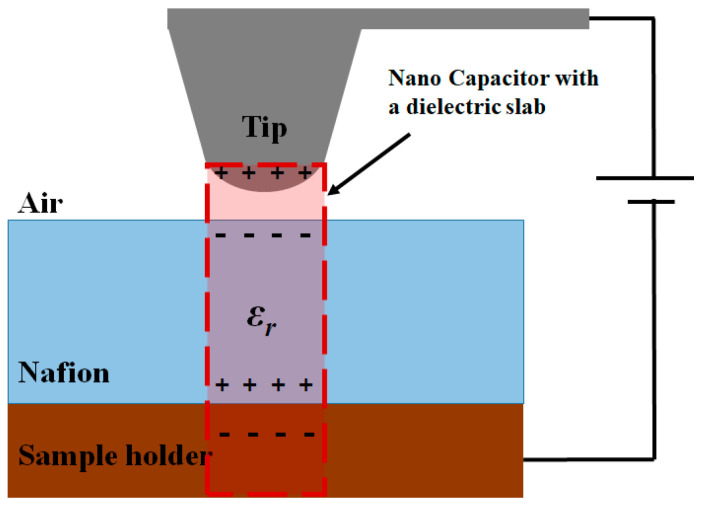
Configuration of the conductive tip and Nafion attached to the sample holder, explaining the origin of the capacitor model.

The tip and sample create a nanosized parallel-plate capacitor filled with air and Nafion, as shown in Figure 1.

The capacitance of this parallel-plate capacitor is calculated as [24,25]

(4) $$C=\frac{Q_{\mathrm{tip}}}{V}$$

A parallel-plate capacitor filled with air and Nafion can be assumed as a dielectric filled capacitor and it can be expressed as [25]

(5) $$V=\frac{Q_{\mathrm{tip}}}{\varepsilon_{0}S}\left(z+\frac{t}{\varepsilon_{r}}\right)$$

where *S* is the area under the tip, *t* is the thickness of the membrane, and *ε_r_* is its relative permittivity. Then,

(6) $$C=\frac{\varepsilon_{0}A}{\left(z+\frac{t}{\varepsilon_{r}}\right)}$$

where *A* is the area of the tip,

(7) $$\frac{\partial C}{\partial z}=-\frac{2\varepsilon_{0}S}{{\left(z+\frac{t}{\varepsilon_{r}}\right)}^{2}}$$

and the capacitance force is

(8) $$F_{C}=-\frac{1}{2}\frac{2\varepsilon_{0}S}{{\left(z+\frac{t}{\varepsilon_{r}}\right)}^{2}}V^{2}$$

Local free charges exist in Nafion due to the ionic domain. Thus, an electrostatic force is also induced between the tip and free charge and is expressed as

(9) $$F_{f}=\frac{1}{4\pi \varepsilon_{0}}\frac{Q_{\mathrm{free}}Q_{\mathrm{tip}}}{z^{2}}=\frac{Q_{\mathrm{free}}}{4z^{2}}R_{\mathrm{tip}}^{2}$$

Hence, the net force between the tip and the sample surface is the sum of the capacitance force of polarized surface and free charges, given as

(10) $$F=-\frac{1}{2}\frac{2\varepsilon_{0}S}{{\left(z+\frac{t}{\varepsilon_{r}}\right)}^{2}}V^{2}+\frac{Q_{\mathrm{free}}}{4z^{2}}R_{\mathrm{tip}}^{2}V$$

and the force gradient is

(11) $$\frac{\partial F}{\partial z}=\frac{\pi \varepsilon_{0}R_{\mathrm{tip}}^{2}}{{\left(z+\frac{t}{\varepsilon_{r}}\right)}^{3}}V^{2}-\frac{Q_{\mathrm{free}}}{2z^{3}}R_{\mathrm{tip}}^{2}V$$

The frequency shift of Nafion is derived by substitution of the net force gradient to the frequency shift [19]

(12) $$∆f≅-\frac{F^{\prime }}{2k}f_{0}=-\frac{\pi \varepsilon_{0}R_{\mathrm{tip}}^{2}}{2k{\left(z+\frac{t}{\varepsilon_{r}}\right)}^{3}}f_{0}V^{2}+\frac{Q_{\mathrm{free}}}{4kz^{3}}R_{\mathrm{tip}}^{2}f_{0}V$$

while the phase shift is given as

(13) $$∆\emptyset ≅A∆f=-\frac{\pi \varepsilon_{0}AR_{\mathrm{tip}}^{2}f_{0}}{2k{\left(z+\frac{t}{\varepsilon_{r}}\right)}^{3}}V^{2}+\frac{Q_{\mathrm{free}}AR_{\mathrm{tip}}^{2}f_{0}}{4kz^{3}}V$$

The variables *k* and *f*_0_ represent the spring constants of a tip and resonance frequency, respectively.

**Figure 2:**
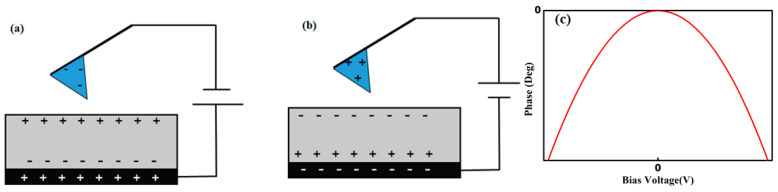
Charge distribution in a system with a (**a**) positive, and (**b**) negative bias voltage applied. (**c**) Variation in phase lag value with bias voltage.

If a sample is uniform and does not contain local surface charges, the polarity of the surface charge of the dielectric sample is opposite to that of the tip charge. Hence, if a positive bias voltage is applied, the tip charge is negative, and the sample surface is positively charged, and vice versa. Thus, the force between the tip and the sample surface is always attractive, as shown in Figure 2, even if the polarity of the bias voltage is changed. In this case, the first term in (13) is dominant. Thus, there is a parabolic relationship between the phase shift and the bias voltage, as shown in Figure 2, which also depends on *ε_r_*. Under identical experimental conditions, if the sample is homogeneous, the phase shift is similar in all scanned areas because the relative permittivity is the same. However, the phase shift changes in a heterogeneous material because of local differences in the relative permittivity. When experimental conditions such as temperature and humidity change, different phase shifts are measured because of these local changes in the relative permittivity.

The behavior of ion exchange membranes can be explained by the combination of the polytetrafluoroethylene (PTFE) backbone and the ionic channel network created by the interconnection of ionic clusters, which consist of sulfonic acid groups. When water binds with the negatively charged sulfonic acid groups, protons are solvated, and free charges exist in the membrane. Since locally charged regions exist in ion exchange membranes, the phase shift is affected by both the first and second terms of (13). Here, *Q_free_* is the local charge related to the ionic cluster; in this case, it is the proton movement into the ionic channel network. The distribution of ionic channel networks on a surface is random, and it changes with surface hydration. Thus, the characterization of ionic clusters in an ion exchange membrane is complicated, and it is even more difficult in composite membranes. However, measuring the force gradient, which is related to free charge, provides a simple quantitative method for characterizing the ionic channel network. Quantitative information on the homogeneity and distribution of the ionic domains on a membrane can be provided by estimating local variations in free charge and relative permittivity.

## 3. Results

**Figure 3:**
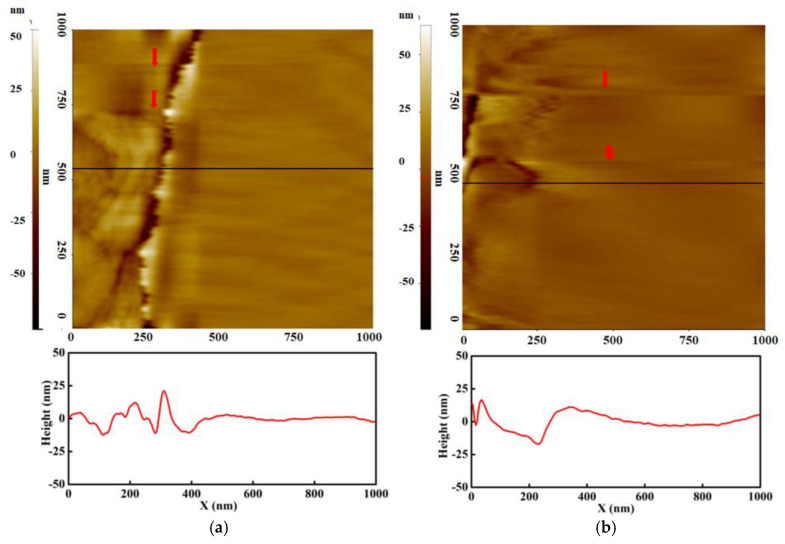
Topography of (**a**) dry and (**b**) wet membrane.

Figure 3 shows the topography and line profile of dry and wet membranes with the bias voltage ranging from −3 V to 3 V in 1 V steps. The brightness in the images indicates height, with brighter (whiter) regions representing higher position. Neither of the membranes show any remarkable structure variation under hydration. Both membranes show smoothly varying surfaces, except the left side of the image. In the wet membrane, the entire surface is smoothly grooved compared with the dry membrane. This is also observed in the line profile; in the wet membrane, the line is gradually curved. It indicates that the morphology of the wet membrane is rougher than the dry membrane. This can also be proved by root mean square (rms) roughness, which can be calculated by the standard deviation of the height variation. The rms roughness of the dry and wet membranes is 12.5 nm and 24.8 nm, respectively. This indicates that the surface becomes rougher after swelling. On top of both images, two blurry lines are observed, which are indicated by red arrows. They indicate the boundary of bias voltage change and are found in EFM images at the same position. Outside the boundary, the morphology does not show any difference. This result implies that applying a bias voltage has an insignificant effect on the topography.

**Figure 4:**
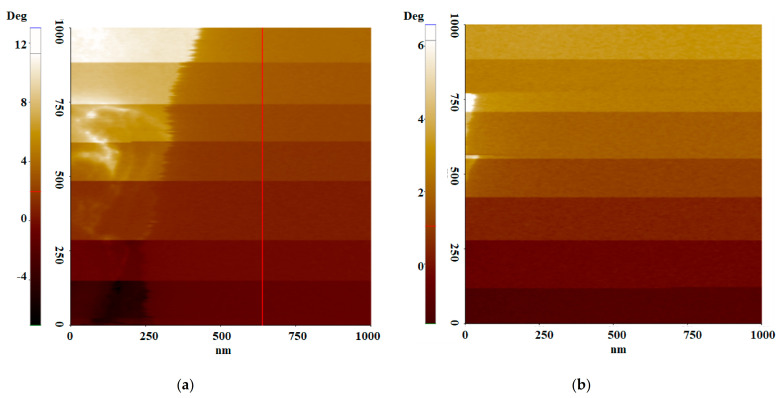
Electrostatic force microscopy (EFM) image of (**a**) dry and (**b**) wet membrane.

Figure 4 depicts the EFM phase images of dry and wet Nafion with the bias voltage ranging from −3 V to 3 V in 1 V steps. The colors in the image indicate the phase lag value, which represents the force gradient. From the image, the color is darker with bias voltage. When the same bias voltage is maintained, the color is uniform except on the left side of the image. This indicates that the areas with homogeneous morphological characteristics have similar phase lag values. The color is brighter from bottom to top of both images. It indicates that the phase lag value is systematically changing. However, the phase lag in each colored region does not follow the parabolic shape that is typical of changes to the force gradient due to induced charge, as shown in Figure 2.

**Figure 5:**
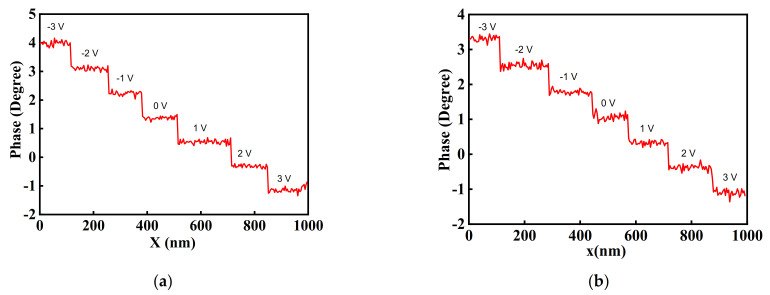
Line profile of (**a**) dry and (**b**) wet membrane.

Figure 5 depicts the line profiles of the dry and wet proton exchange membranes, providing numerical information on the phase shift at each bias voltage. Both images show small changes for a phase shift of ~0.2° when the same bias voltage is maintained, and a relatively large phase shift of 1° is observed when the bias voltage changes. Both membranes have positive phase shift values between −3 V and 0 V, indicating that the net electrostatic force between the tip and the sample surface is repulsive. In the negative bias voltage configuration, the tip is positively charged, and typically, the force between the tip and the sample surface is attractive, owing to the negatively polarized membrane surface. The result depicts the opposite phenomenon, implying that the sample surface is positively charged. For negative bias voltages, phase lag values are slightly higher for dry membranes than those for wet membranes. The phase shift is negative between 2 V and 3 V, indicating that the force is in the attractive regime. With these bias voltages, both membranes have similar phase lag values.

**Figure 6:**
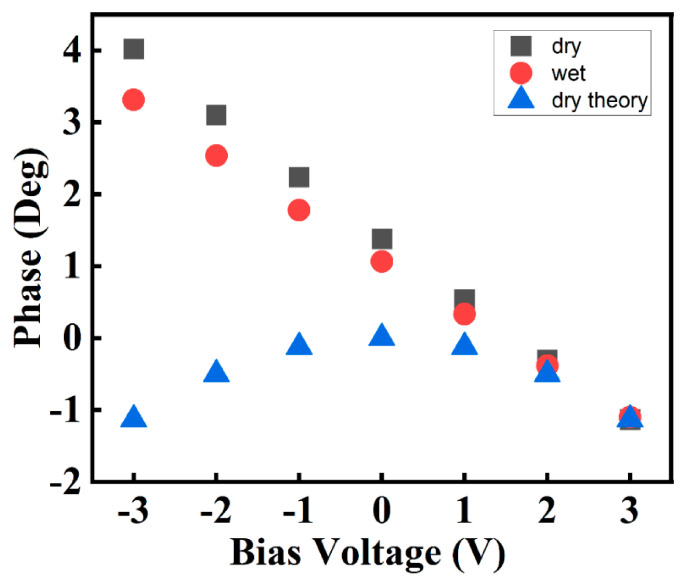
Variation in phase lag with bias voltage for different membrane conditions.

For more detailed analysis, the mean phase value at each bias voltage was plotted for both the dry and wet membranes. From the analysis, it can be observed that the phase lag value varies linearly with bias voltage in both membranes, as shown in Figure 6. There are locally charged regions on the membrane, the behavior of which is characterized by the second term in (13). As the phase lag is the sum of both terms in (13), a positive phase lag value indicates that the second term, related to the local surface charge, is dominant. When the bias voltage is reduced, the phase lag decreases. For the wet membrane, the phase lag values of 3.4°, 2.5°, and 1.8° were noted at bias voltages of −3 V, −2 V, and −1 V, indicating that both terms in the equation decreased as the bias voltage was reduced. For the dry membrane, the phase lag values were 4.0°, 3.4°, and 2.5° at −3 V, −2 V, and −1 V, respectively. Wet membranes typically have higher proton conductivities than dry membranes, and a high ionic channel network density, because of their creation of a new ionic channel network. The difference between the phase lag values of wet and dry membranes is thus related to the second term in (13). At 1 V, this value is close to zero. In contrast, at 2 V and 3 V, both membranes have similar negative phase values. Both membranes have similar phase values at 2 V and 3 V. Specifically, dry and wet membranes have the same lag values at 3 V. This result implies that the electrical interaction is only between the charged tip and the polarized surface charge. Thus, the second term in (13) does not have any effect on the phase lag in this case.

## 4. Analysis

**Figure 7:**
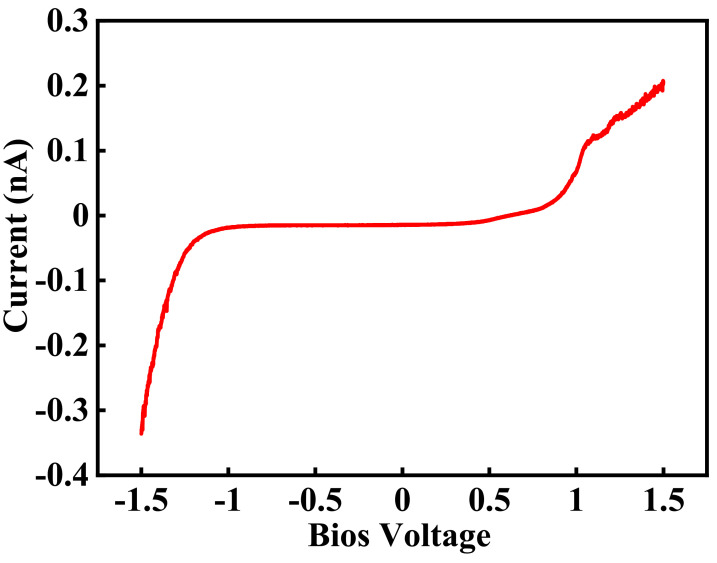
Variation in current with bias voltage applied to the Pt tip.

Local charge density, which reflects the ionic channel network, can be approximated based on the first and second terms of (13). For this, the phase lag value at each bias voltage must be related to a microscopic electrostatic phenomenon. To understand the generation of positive phase lag at a negative sample bias voltage, the operation of a tip when bias voltage is applied during scanning must be analyzed. There is typically a water layer between the tip and the sample surface. When a bias voltage is applied, hydrolysis occurs, hydrogen is produced, and protons are created because of the Pt-coated tip. Figure 7 depicts the local variation in the current flowing through the Pt tip, and the half membrane electrode assembly as bias voltage is swept. Current flows when the magnitude of the bias voltage is larger than 1.5 V, indicating that protons are created when a voltage is applied to the Pt tip.

**Figure 8:**
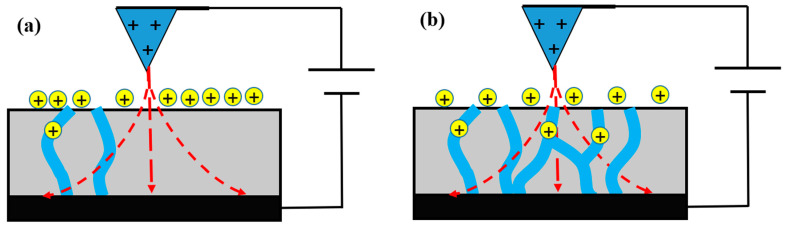
Proton movement into the ionic channel with (**a**) negative and (**b**) positive sample bias voltages.

The phase lag generated at negative bias voltages includes a contribution from the interaction between the released protons and the ionic domains on the membrane surface. As the membrane is negatively charged, owing to polarization, it attracts protons that cover its surface. Thus, positive phase lag values are measured, because a repulsive force is induced between the positively charged tip and the proton-covered surface. The magnitude of the repulsive force is related to the density of the activated ionic channel network. When water uptake in the membrane increases, an ionic channel network is developed, as the number of interconnections between the ionic channels grows. Protons are accelerated into the ionic channel by the external electric field, as shown in Figure 8. The number of ionic domains increases as the number of protons on the membrane surface decreases. Thus, the repulsive force between the tip and the membrane and the area of the ionic domain have a reciprocal relationship.

Table 1 lists the mean phase lag values for dry and wet membranes, and the values when there are no protons on the membrane surface. The latter values were calculated using only negative bias voltages. With both dry and wet membranes, the phase lag increased with the bias voltage, which can be explained as the increase in proton generation due to hydrolysis. At all negative bias voltages, dry membranes have a larger phase lag value than wet membranes, which is consistent with our assumptions. Hence, the area of the ionic domain on the membrane can be approximated using a phase lag difference.

The net electrical charge of the protons at each bias voltage and membrane condition was estimated using (13). This approximation is conducted in several steps. First, because the phase lag value obtained for each membrane includes a contribution from the polarization-induced charge, the phase lag when there are no protons on the membrane surface is subtracted from this value. Then, the tip radius is calculated for each membrane using the blind tip reconstruction method [26]. Finally, the net charge of the protons is calculated using the second term of (13).

The calculation results for the net charge are summarized in Table 2. In the dry membrane, the net charge is 8.71 × 10^−18^ C, 6.07 × 10^−18^ C, and 3.99 × 10^−18^ C at −3 V, −2 V, and −1 V, respectively. Hence, the net charge increases as the bias voltage is increased. The value at −1 V is much smaller than the net charge at other voltages, owing to the relatively small amount of proton generation at −1 V. This is consistent with the variation in local current with a swept bias voltage. However, the latter result does not provide absolute numerical information about the ionic domain. In the wet membrane, the net charge is 1.87 × 10^−18^ C, 1.28 × 10^−18^ C, and 8.06 × 10^−18^ C at −3 V, −2 V, and −1 V, respectively. This trend is similar to that observed for dry membranes. However, the amount of electrical charge is much smaller than that with dry membranes, possibly because of the partial movement of protons into the ionic channels. This result indicates that wet membranes have a larger ionic domain than dry membranes. Here, the repulsive force is only due to the protons that do not move into the ionic channel network. The difference between the net charge of dry and wet membranes is similar at each bias voltage and is ~79–80%. This result implies that 80% of the liberated protons move into the wet membrane; only 20% of the protons interact with the tip, and this ratio is independent of the bias voltage. From these results, it can be surmised that the area of the ionic channels on the surface of a wet membrane increases by ~80% compared with that on a dry membrane. Previous experimental results have shown that there is an approximately 80% difference between proton conductivity under ambient conditions and fully humid conditions [27,28]. Hence, our calculations are consistent with the literature.

In this study, we derived an NAM for analyzing proton exchange membranes. Based on Shen’s study [19], we used an interpretation method for EFM signals. We assumed that the capacitive force is a summation of two dominant electrostatic interactions: electrostatic force of induced charge-charged tip and free charge-charged tip. We derived the force gradient, which was recorded as the phase lag value on the EFM image, based on these two interactions. Thus, the NAM considers two terms: the polarization dominant term and the free charge dominant term. The backbone is ruled by the polarization dominant term, and the free charge dominant term is related to the ionic domain structure of proton exchange membranes. Thus, the structural change of the ionic domain can be characterized by adapting the NAM to measure the phase lag value of EFM. To examine the NAM, we determined the local charge density of a proton exchange membrane, which is directly related to the ionic domain, by using an approximation model. The wet and dry Nafion was scanned by increasing the applied bias voltage in intervals and applying protons from hydrolysis. The characterization by the NAM charge density of protons on the surface shows a clear difference between the dry and wet membranes. The results are in good agreement with those of previous studies [21]. Thus, we conclude that the NAM can be applied for studying proton exchange membranes. The enhancement of proton conductivity is the prime purpose for developing the proton exchange membranes. Proton conductivity is governed by the morphological structure of the ionic channel network. Thus, the characterization of the ionic channel network is mandatory for developing the novel proton exchange membranes. The NAM for local charge density derived using electrostatic force microscopy has become an important tool for characterizing a novel proton exchange membrane.

## 5. Conclusions

In this study, we proposed a NAM that focusses on free charge movement into the ionic channel network of a proton exchange membrane, based on the capacitance force between a conductive AFM tip and the proton exchange membrane surface. The model is expressed as a summation of induced charge distribution, which is connected with the backbone of the proton exchange membrane and free charge distribution which is related to the ionic channel network. This model can be used for ionic channel network variation under various conditions, such as hydration, as well as for composites with filler materials, by calculating induced and free charge distribution change. The NAM was verified by analysis of the experimental results, which were phase lags measured under different bias voltages for dry and wet proton exchange membranes.

The enhancement of proton conductivity is the prime purpose of developing proton exchange membranes. Proton conductivity is governed by the morphological structure of ionic channel networks. Thus, the characterization of ionic channel networks is essential for developing novel proton exchange membranes. The NAM is shown to be an important tool for characterizing novel proton exchange membranes.

## Figures and Tables

**Table 1:** Mean phase lag value of each membrane.

Bias Voltage (V)	Dry Membrane (Degree)	Wet Membrane (Degree)	No. Protons (Degree)	Phase Lag Difference between Wet Membrane and No. Protons	Phase Lag Difference between Dry Membrane and No. Protons
−3	4.02	3.31	−1.134	5.15	4.45
−2	3.10	2.54	−0.504	3.60	3.04
−1	2.24	1.78	−0.126	2.36	1.90

**Table 2:** Net charge of each membrane.

Bias Voltage (V)	Net Charge of Dry Membrane (C)	Net Charge of Wet Membrane (C)	% Difference
−3	8.71 × 10^−18^	1.87 × 10^−18^	78.5
−2	6.07 × 10^−18^	1.28 × 10^−18^	78.9
−1	3.99 × 10^−18^	0.81 × 10^−18^	79.8

## Data Availability

Data are contained within the article.

## References

[1] Zuo Z.C., Fu Y.Z., Manthiram A. (2012). Novel Blend Membranes Based on Acid-Base Interactions for Fuel Cells. Polymers.

[2] Ketpang K., Lee K., Shanmugam S. (2014). Facile Synthesis of Porous Metal Oxide Nanotubes and Modified Nafion Composite Membranes for Polymer Electrolyte Fuel Cells Operated under Low Relative Humidity. ACS Appl. Mater. Inter..

[3] Zhu J., Tang H.L., Pan M. (2008). Fabrication and characterization of self-assembled Nafion-SiO_2_-ePTFE composite membrane of PEM fuel cell. J. Membrane Sci..

[4] Ke C.C., Li X.J., Shen Q.A., Qu S.G., Shao Z.G., Yi B.L. (2011). Investigation on sulfuric acid sulfonation of in-situ sol-gel derived Nafion/SiO_2_ composite membrane. Int. J. Hydrogen. Energy.

[5] Hsu W.Y., Gierke T.D. (1983). Ion-Transport and Clustering in Nafion Perfluorinated Membranes. J. Membrane Sci..

[6] Schmidt-Rohr K., Chen Q. (2008). Parallel cylindrical water nanochannels in Nafion fuel-cell membranes. Nat. Mater..

[7] Gupit C.I., Li X., Maekawa R., Hasegawa N., Iwase H., Takata S., Shibayama M. (2020). Nanostructures and Viscosities of Nafion Dispersions in Water/Ethanol from Dilute to Concentrated Regimes. Macromolecules.

[8] Park H.S., Kim Y.J., Hong W.H., Choi Y.S., Lee H.K. (2005). Influence of morphology on the transport properties of perfluorosulfonate ionomers/polypyrrole composite membrane. Macromolecules.

[9] Duvigneau J., Schonherr H., Vancso G.J. (2010). Nanoscale Thermal AFM of Polymers: Transient Heat Flow Effects. ACS Nano..

[10] Sen S., Subramanian S., Discher D.E. (2005). Indentation and adhesive probing of a cell membrane with AFM: Theoretical model and experiments. Biophys. J..

[11] Xie X., Kwon O., Zhu D.M., Van Nguyen T., Lin G.Y. (2007). Local probe and conduction distribution of proton exchange membranes. J. Phys. Chem. B.

[12] Palermo V., Palma M., Samori P. (2006). Electronic characterization of organic thin films by Kelvin probe force microscopy. Adv. Mater..

[13] Dugger J.W., Collins L., Welbourn R.J.L., Skoda M.W.A., Balke N., Lokitz B.S., Browning J.F. (2018). Ion movement in thin Nafion films under an applied electric field. Appl. Phys. Lett..

[14] Girard P. (2001). Electrostatic force microscopy: Principles and some applications to semiconductors. Nanotechnology.

[15] Zhao J.W., Uosaki K. (2003). Dielectric properties of organic monolayers directly bonded on silicon probed by current sensing atomic force microscope. Appl. Phys. Lett..

[16] Melin T., Diesinger H., Deresmes D., Stievenard D. (2004). Electric force microscopy of individually charged nanoparticles on conductors: An analytical model for quantitative charge imaging. Phys. Rev. B.

[17] Han B., Chang J.X., Song W., Sun Z., Yin C.Q., Lv P.H., Wang X. (2019). Study on Micro Interfacial Charge Motion of Polyethylene Nanocomposite Based on Electrostatic Force Microscope. Polymers.

[18] Deschler J., Seiler J., Kindersberger J. (2017). Detection of Charges at the Interphase of Polymeric Nanocomposites. IEEE Trans. Dielect. Electr. Insul..

[19] Shen Y., Wang Y., Zhou Y., Hai C.X., Hu J., Zhang Y. (2018). Electrostatic force spectroscopy revealing the degree of reduction of individual graphene oxide sheets. Beilstein. J. Nanotech..

[20] Wang P., Olbricht W.L. (2010). PEDOT/Nafion composite thin films supported on Pt electrodes: Facile fabrication and electrochemical activities. Chem. Eng. J..

[21] Wang Z.B., Tang H.L., Li J.R., Jin A.P., Wang Z., Zhang H.J., Pan M. (2013). Balancing dimensional stability and performance of proton exchange membrane using hydrophilic nanofibers as the supports. Int. J. Hydrogen. Energy.

[22] Barnes A.M., Buratto S.K. (2018). Imaging Channel Connectivity in Nafion Using Electrostatic Force Microscopy. J. Phys. Chem. B.

[23] Barnes A.M., Du Y.F., Liu B., Zhang W.X., Seifert S., Coughlin E.B., Buratto S.K. (2019). Effect of Surface Alignment on Connectivity in Phosphonium-Containing Diblock Copolymer Anion-Exchange Membranes. J. Phys. Chem. C.

[24] Lilliu S., Maragliano C., Hampton M., Elliott M., Stefancich M., Chiesa M., Dahlem M.S., Macdonald J.E. (2013). EFM data mapped into 2D images of tip-sample contact potential difference and capacitance second derivative. Sci. Rep..

[25] Griffiths D.J. (2005). Introduction to Electrodynamics. Am. J. Phys..

[26] Flater E.E., Zacharakis-Jutz G.E., Dumba B.G., White I.A., Clifford C.A. (2014). Towards easy and reliable AFM tip shape determination using blind tip reconstruction. Ultramicroscopy.

[27] Son B., Oh K., Park S., Lee T.G., Lee D.H., Kwon O. (2019). Study of morphological characteristics on hydrophilicity-enhanced SiO_2_/Nafion composite membranes by using multimode atomic force microscopy. Int. J. Energy Res..

[28] Kim A.R., Vinothkannan M., Lee K.H., Chu J.Y., Ryu S.K., Kim H.G., Lee J.Y., Lee H.K., Yoo D.J. (2020). Ameliorated Performance of Sulfonated Poly(Arylene Ether Sulfone) Block Copolymers with Increased Hydrophilic Oligomer Ratio in Proton-Exchange Membrane Fuel Cells Operating at 80% Relative Humidity. Polymers.

